# Supplementary material for: Fast detection of synergy and antagonism in antifungal combinations used against Candida albicans clinical isolates
Source: Sci Rep. 2025 Oct 15;15:36103. doi: 10.1038/s41598-025-22870-x (PMC12528425; doi:10.1038/s41598-025-22870-x)
Supplement: Supplementary file 1 — Supplementary Material 1 [file 41598_2025_22870_MOESM1_ESM.docx]

## Supplementary information

**Supplementary protocol 1:** **Checkerboard protocol for antifungal combinations**

Protocol has been adapted from E. Dannouli et al and EUCAST protocol for MIC determination of yeasts by broth microdilution. The RPMI medium used throughout the protocol is supplemented with 2% glucose

1. Grow the fungal isolates of interest on YPD plates overnight at 30°C.
2. Prepare antifungal stocks in RPMI medium at 4X the highest concentration to be used in the assay.
3. Perform a 2-fold serial dilution in RPMI medium of each antifungal stock prepared, yielding a 4X dilution of all antifungal concentrations to be used.
4. Drug A is added to rows B-H. Add 50 ul of the prepared dilutions to each well with the highest concentration in row B and lowest concentration in row H to a 96-well round bottom microtiter plate.
5. Drug B is added to columns 2-11. Add 50 ul of the prepared dilutions to each well with the highest concentration in column 2 and lowest concentration in column 11.
   1. Based on step 3-4, wells B-H in column 1 contains only drug A and wells 2-11 in row A only drug B. Add 50 ul of RPMI medium to these wells for a final volume of 100 ul.
6. Prepare the fungal inoculum by suspending colonies in RPMI medium until a turbidity of 0.5 McFarland.
7. Add 100 ul of the fungal inoculum to all wells in column 1-11.
8. Well A1 serves as the growth control. Add 100 ul of RPMI medium to the 100 ul of inoculum
9. Well A12 serves as the sterility control. Add 200 ul of RPMI medium.
10. Incubate plate at 30°C overnight.
    1. To reduce evaporation, plates can be placed in plastic bags prior to incubation.
11. Analyse plates by measuring the OD at 450 nm
    1. If needed, resuspend wells prior to the OD measurement
12. Ensure sterility by comparing the sterility control with the empty wells in column 12.

**Supplementary protocol 2: Protocol for antifungal combination testing using combination plates**

**Day 1:**

1. Grow chosen strains in 2 ml of Yeast-Peptone-Dextrose (YPD) medium at 30°C in an orbital shaker overnight (ON).
   1. Places tubes diagonally in rack.
2. Prepare YPD agar according to manufacturer’s instruction, keep at 55°C.
3. Prepare a 1 mg/mL of methylene blue in sterile dH_2_O. Shield from direct light and store at 4°C until use.

**Day 2:**

1. Dilute antifungal stock to the desired input concentration in YPD agar. Keep antifungal-agar in a water bath set at 55°C until use.
2. Load 0.5 mL of the antifungal agar to the assigned chamber of the CombiANT assay
   1. Ensure entire chamber is filled and remove any air bubbles.
3. Let agar solidify at room temperature, then keep at 4°C for at least 45 minutes until further use.
4. To activate the assay, remove plates from 4°C and add a 25 mL underlay of YPD agar to each plate.
   1. Add agar centrally of the assay, ensure all chambers are submerged and pipette away any bubbles.
   2. Do not move the plates until agar has solidified as it may disrupt the diffusion pattern
5. Let the agar set for at least 2 hours at room temperature after the last plate is cast
6. Prior to plating of the strains, prepare n + 1 mL (n = number of plates) of low-gelling temperature agarose at 3% (w/v) in sterile dH_2_O. Melt and keep agar at 55°C in a water bath.
   1. Add 1 μL of the methylene blue solution per mL of low-gelling temperature agarose solution prepared.
7. Dilute the ON cultures of the chosen strains 1:50 in YPD medium.
8. Per plate, mix 1 mL of the diluted ON cultures with 1 mL of the low-gelling temperature agarose with methylene blue. Mix by pipetting and pour immediately onto the plate.
   1. Ensure entire plate is covered by the overlay.
9. Let overlay solidify at room temperature for 10 min, then incubate ON at 30°C.

**Day 3.**

1. Take pictures of the plates. Orient plates so chamber A is furthest down for easier analysis.
2. Identify the points of interest using the CombiANT Imager software (Rx Dynamics AB).
3. Input the data into the analysis algorithm.
